# The golgin GMAP-210 is required for efficient membrane trafficking in the early secretory pathway

**DOI:** 10.1242/jcs.166710

**Published:** 2015-04-15

**Authors:** Peristera Roboti, Keisuke Sato, Martin Lowe

**Affiliations:** Faculty of Life Sciences, University of Manchester, The Michael Smith Building, Oxford Road, Manchester M13 9PT, UK

**Keywords:** Golgi, Golgin, Membrane traffic, Secretion, ERGIC

## Abstract

Golgins are coiled-coil proteins that participate in membrane-tethering events at the Golgi complex. Golgin-mediated tethering is thought to be important for vesicular trafficking and Golgi organization. However, the degree to which individual golgins contribute to these processes is poorly defined, and it has been proposed that golgins act in a largely redundant manner. Previous studies on the golgin GMAP-210 (also known as TRIP11), which is mutated in the rare skeletal disorder achondrogenesis type 1A, have yielded conflicting results regarding its involvement in trafficking. Here, we re-investigated the trafficking role of GMAP-210, and found that it is indeed required for efficient trafficking in the secretory pathway. GMAP-210 acts at both the endoplasmic reticulum (ER)-to-Golgi intermediate compartment (ERGIC) and Golgi complex during anterograde trafficking, and is also required for retrograde trafficking to the ER. Using co-depletion experiments, we also found that GMAP-210 acts in a partially redundant manner with the golgin GM130 to ensure efficient anterograde cargo delivery to the cis-Golgi. In summary, our results indicate a role for GMAP-210 in several trafficking steps at the ER–Golgi interface, some of which are partially redundant with another golgin, namely GM130 (also known as GOLGA2).

## INTRODUCTION

The Golgi complex lies at the heart of the secretory pathway where it acts as a processing factory and sorting hub for cargo proteins and lipids (Rothman, 1981). The vertebrate Golgi complex comprises stacked cisternae that are laterally linked to form the Golgi ribbon, which is usually found in close proximity to the centrosome (Lowe, 2011; Shorter and Warren, 2002). Many proteins have been implicated in secretory trafficking and Golgi organization, and among these are the golgin family, of which there are at least 11 members in vertebrates (Munro, 2011). Golgins are predominantly coiled-coil proteins that are typically anchored to the cytosolic face of the Golgi membrane by their extreme C-termini (Munro, 2011; Barinaga-Rementeria Ramirez and Lowe, 2009). These features have led to the proposal that golgins extend into the cytoplasm to capture, or tether, transport vesicles to Golgi membranes, which can be followed by membrane fusion in order to facilitate both membrane traffic and assembly or maintenance of Golgi cisternae (Munro, 2011; Barinaga-Rementeria Ramirez and Lowe, 2009). Tethering could also occur between Golgi membranes to promote stacking or Golgi ribbon formation.

Evidence for golgin-mediated tethering exists (Wong and Munro, 2014), but the extent to which different golgins participate in secretory trafficking remains poorly defined. Several studies have shown defective trafficking upon loss of individual golgins (Brémond et al., 2009; Diao et al., 2008; Diao et al., 2003; Hicks et al., 2006; Koreishi et al., 2013; Lieu et al., 2008; Yamane et al., 2007), although the effects are usually subtle, whereas others have failed to observe any consequences upon secretory trafficking (Friggi-Grelin et al., 2006; Puthenveedu et al., 2006; Vasile et al., 2003; Yadav et al., 2009). These findings have led to the proposal that golgins function in a redundant manner, acting collectively on the surface of Golgi membranes to tether vesicles and Golgi membranes (Munro, 2011; Sinka et al., 2008). In this model, the loss of a single golgin is compensated for by other golgins with a similar tethering specificity.

GMAP-210 (also known as TRIP11) is a golgin localized to the cis-Golgi (Cardenas et al., 2009; Rios et al., 1994). It is anchored to Golgi membranes by a C-terminal GRIP-related Arf-binding (GRAB) domain and is able to tether vesicles through an amphipathic-lipid-packing sensor (ALPS) motif present at the extreme N-terminus (Drin et al., 2008; Gillingham et al., 2004). Several studies have investigated the cellular function of GMAP-210. Depletion of GMAP-210 in tissue culture cells has been reported to cause Golgi ribbon fragmentation, whereas secretory trafficking and Golgi stack organization were unaffected (Ríos et al., 2004; Yadav et al., 2009). These observations suggested that GMAP-210 is not required for trafficking. By contrast, overexpression of GMAP-210 in human and *Drosophila* cells has been found to cause Golgi vesiculation accompanied by a block in secretory trafficking (Friggi-Grelin et al., 2006; Pernet-Gallay et al., 2002), although these effects could be indirect, possibly through sequestration of important binding partners. GMAP-210-knockout mice also display Golgi vesiculation and impaired cargo secretion, but this is evident only in certain cell types, most notably chondrocytes, which are responsible for cartilage and bone deposition (Smits et al., 2010). The physiological importance of GMAP-210 is indicated by the fact that mutations in human GMAP-210 cause the neonatal lethal skeletal dysplasia achondrogenesis type 1A (Smits et al., 2010), although whether this arises from altered glycosylation and/or reduced secretion of extracellular matrix proteins remains to be ascertained (Smits et al., 2010). Additional functions for GMAP-210 have also been proposed, including linking the Golgi complex to the centrosome (Ríos et al., 2004), and anchoring IFT20 to the Golgi complex, which might be important for ciliogenesis (Broekhuis et al., 2013; Follit et al., 2008).

There is therefore conflicting evidence regarding the extent to which GMAP-210 participates in secretory trafficking. It is also unclear which trafficking steps GMAP-210 may participate in, and whether its role in trafficking is redundant with other golgins. In this study we report that GMAP-210 is required for efficient anterograde and retrograde trafficking in the early secretory pathway, functioning at both the ER-to-Golgi intermediate compartment (ERGIC) and Golgi complex. We also report partial functional overlap with the golgin GM130 (also known as GOLGA2), supporting the view that golgins can act, at least partially, in a redundant capacity to ensure efficient membrane traffic.

## RESULTS

### GMAP-210 is required for efficient ER-to-Golgi trafficking

**Fig. 1.**
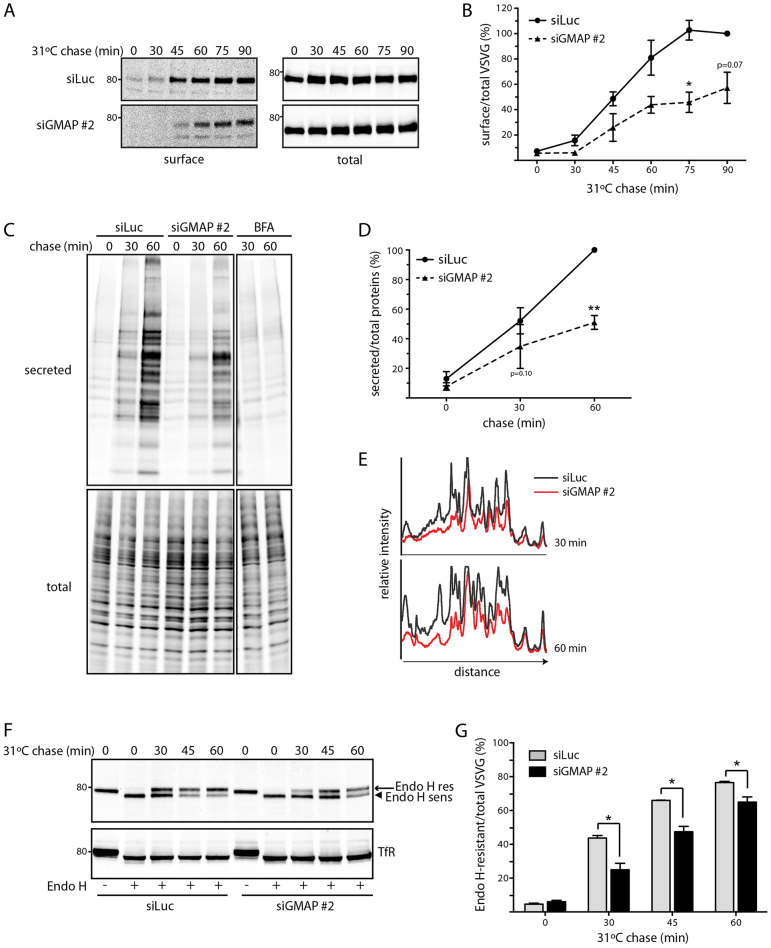
**GMAP-210 depletion decreases secretory trafficking.GMAP-210 depletion decreases secretory trafficking.** (A) HeLa M cells transfected with control siRNA targeting luciferase (siLuc) or siRNA against GMAP-210 (siGMAP #2) were infected with ts045-VSVG–GFP adenovirus, and incubated at 40°C for 16 h to retain misfolded VSVG in the ER. The cells were then shifted to 31°C for the indicated time periods (chase) to induce trafficking. Surface proteins were biotinylated and isolated using streptavidin beads. Streptavidin precipitates (surface) and total cell lysates (total) were assayed by western blotting with anti-GFP antibody. (B) The ratio of surface-to-total VSVG at the indicated time points in A is expressed as a percentage of the value in control siLuc-treated cells at 90 min. Data show the mean±s.e.m from three independent experiments. The *P*-values are calculated against the matched time points in the control siLuc-treated cells. *P<0.05 (paired Student's *t*-test). (C) HeLa M cells were transfected with the indicated siRNAs or treated with BFA at 5 µg/ml for 1 h. Newly synthesized proteins were radiolabeled for 10 min and then chased in medium containing unlabeled Met and Cys for the indicated times (chase). BFA was included throughout the pulse and chase in BFA-treated cells. At each time, the medium was collected and secreted proteins were precipitated with TCA, whereas cells were lysed. Radiolabeled proteins in TCA precipitates (secreted) and total cell lysates (total) were resolved by SDS-PAGE and visualized by phosphorimaging. (D) The ratio of secreted-to-total proteins at the indicated time points in C was calculated and is expressed as a percentage of the value in siLuc-treated cells at 60 min. Data represent mean±s.e.m from three independent experiments. *P<0.05, **P<0.01 (paired Student's *t*-test). (E) Line intensity scans of signals from [^35^S]-labeled secreted proteins. (F) HeLa M cells transfected with the indicated siRNAs were infected with ts045-VSVG–GFP, and incubated at 40°C for 16 h. Cells were shifted to 31°C for the indicated time period (chase), and lysed. Proteins were treated with Endo H (Endo H +) and analyzed by western blotting with anti-GFP antibody. Bands corresponding to Endo-H-sensitive (arrowhead, ER form) and Endo-H-resistant (arrow, Golgi form) VSVG–GFP are indicated. (G) Quantitation of F to indicate the percentage of VSVG–GFP in Endo-H-resistant form. Data represent mean±s.e.m from three independent experiments. *P<0.05 (paired Student's *t*-test).

To investigate whether GMAP-210 is required for secretory trafficking, we used the well-characterized model cargo protein ts045-VSVG, a temperature sensitive mutant of the vesicular stomatitis virus glycoprotein. This model cargo accumulates in the ER at the non-permissive temperature (40°C), and transits the secretory pathway in a synchronous manner when shifted to the permissive temperature of 31°C (Kreis and Lodish, 1986). Two groups have previously reported that GMAP-210 is dispensable for the trafficking of ts045-VSVG, as assessed by immunofluorescence microscopy (Smits et al., 2010; Yadav et al., 2009). However, it is conceivable that a delay in trafficking might have been missed using this method of analysis. We therefore used a more quantitative biochemical approach to monitor trafficking of ts045-VSVG after depletion of GMAP-210. Using surface biotinylation to measure delivery to the plasma membrane, we observed reduced transport of ts045-VSVG–GFP from the ER to the cell surface in cells in which GMAP-210 was depleted using small interfering RNA (siRNA, denoted siGMAP) (Fig. 1A,B). A similar reduction was observed using a second siRNA, as well as a mixture of four siRNAs targeting GMAP-210, confirming the specificity of the effect (supplementary material Fig. S1A,B). Consistent with delayed trafficking to the plasma membrane, the secretion of newly synthesized proteins into the medium was reduced in GMAP-210-depleted cells (Fig. 1C,D). Interestingly, the profile of secreted proteins was affected by GMAP-210 depletion, suggesting that trafficking of some cargo proteins is more sensitive to loss of GMAP-210 than others (Fig. 1E).

We next monitored transport from the ER to the Golgi complex, using the acquisition of endoglycosidase H (Endo H) resistance as a measure for delivery to the medial Golgi. There was delayed acquisition of Endo H resistance in the GMAP-210-depleted cells (Fig. 1F,G). A similar delay was observed with three independent siRNAs and a mixture of four siRNAs targeting GMAP-210 (supplementary material Fig. S1C-E). Although we cannot exclude an effect of GMAP-210 depletion upon Endo H sensitivity through alteration of Golgi enzyme distribution, the kinetic delay in Endo H resistance (Fig. 1F,G; supplementary material Fig. S1C-E) is similar to that seen for surface biotinylation (Fig. 1A,B; supplementary material Fig. S1A,B), suggesting it is primarily a defect in trafficking that is responsible (see also below).

### GMAP-210 functions in anterograde trafficking to both the ERGIC and Golgi complex

**Fig. 2.**
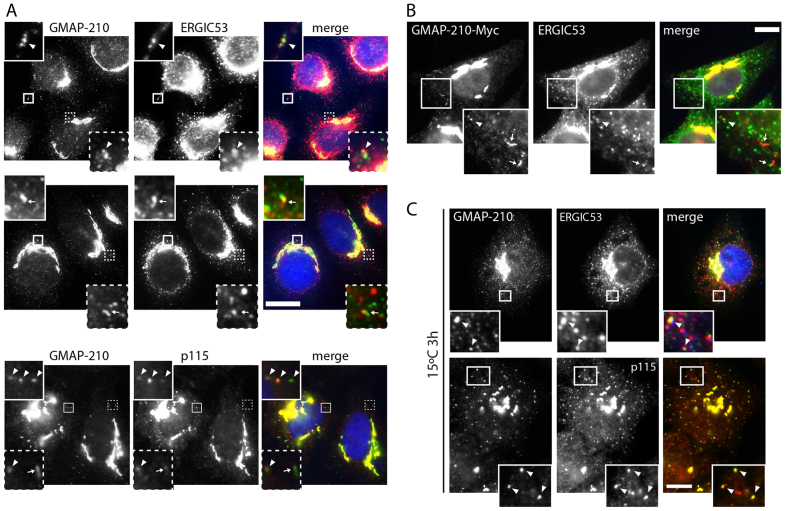
**GMAP-210 localizes to the ERGIC as well as the cis-Golgi.GMAP-210 localizes to the ERGIC as well as the cis-Golgi.** (A) HeLa cells were stained for endogenous GMAP-210 (green) and ERGIC53 or p115 (red) followed by immunofluorescence microscopy. The insets show magnified views of the selected areas. Arrowheads indicate puncta positive for GMAP-210 and ERGIC53 or p115. Arrows indicate GMAP-210 tubules originating from ERGIC53 or p115 puncta. (B) HeLa cells transfected with full-length GMAP-210–Myc were stained for the Myc epitope (green) and endogenous ERGIC53 (red). In the inset, arrowheads indicate puncta positive for GMAP-210 and ERGIC53, and arrows indicate GMAP-210 tubules originating from ERGIC53 puncta. (C) HeLa cells were incubated at 15°C for 3 h before staining for endogenous GMAP-210 (green) and ERGIC53 or p115 (red). Insets highlight the localization of GMAP-210 at peripheral ERGIC53- and p115-positive structures, indicated by arrowheads. Scale bars: 10 µm.

We next wanted to identify the trafficking step(s) between the ER and Golgi apparatus that GMAP-210 is involved in. Previous work has localized GMAP-210 at steady state to the ERGIC and cis-Golgi (Rios et al., 1994). GMAP-210 is also recruited to the ERGIC upon treatment with brefeldin A (BFA) (Rios et al., 1994), suggesting it cycles into earlier compartments of the secretory pathway. Consistent with this possibility, in untreated cells, we detected both endogenous and ectopically expressed GMAP-210 in cytoplasmic puncta that partially overlapped with the ERGIC markers ERGIC53 and p115 (Fig. 2A,B). Interestingly, many of the peripheral structures were short tubules, and they sometimes appeared to emanate from ERGIC elements (Fig. 2A,B). Upon shifting to 15°C to arrest trafficking to the Golgi complex (Saraste and Kuismanen, 1984), there was enhanced GMAP-210 localization at the ERGIC (Fig. 2C). These results are consistent with a pool of GMAP-210 localizing to the ERGIC, suggesting that it might participate in trafficking at this compartment in addition to the Golgi complex.

**Fig. 3.**
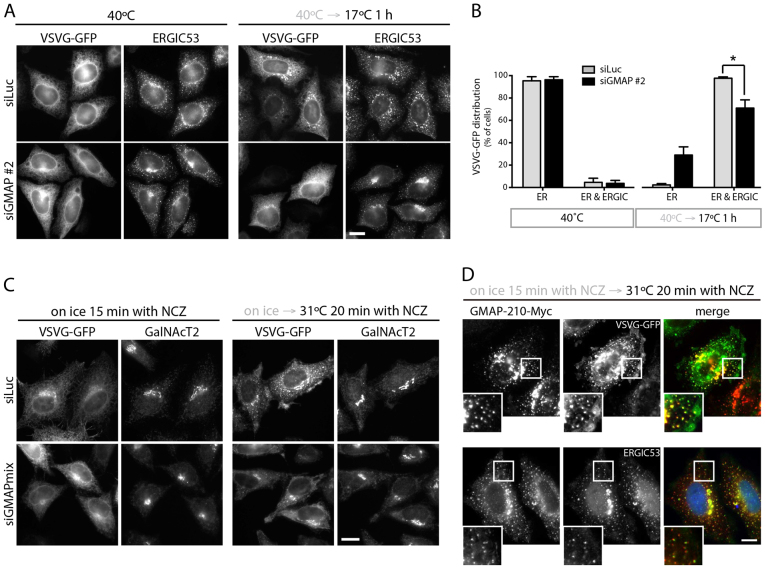
**The trafficking of VSVG–GFP to pre-Golgi structures is impaired by depletion of GMAP-210.The trafficking of VSVG–GFP to pre-Golgi structures is impaired by depletion of GMAP-210.** (A) Luciferase (siLuc) or GMAP-210 (siGMAP #2)-depleted HeLa cells were infected with ts045-VSVG–GFP and incubated at 40°C for 16 h. Cells were shifted to 17°C for 1 h to induce VSVG–GFP accumulation in the ERGIC prior to staining for endogenous ERGIC53. (B) Quantification of VSVG–GFP localization (50–100 cells per condition) from A. Data show mean±s.e.m from three independent experiments. *P<0.05 (paired Student's *t*-test). (C) HeLa cells transfected with the indicated siRNAs were infected with ts045-VSVG–GFP adenovirus, and incubated at 40°C for 16 h. Cells were then placed on ice for 15 min with nocodazole (NCZ), and either fixed directly, or warmed to 31°C in the presence of the drug for 20 min before staining for endogenous GalNAcT2. (D) HeLa cells expressing GMAP-210–Myc and ts045-VSVG–GFP were placed on ice for 15 min with NCZ. Cells were shifted to 31°C for 20 min in the presence of NCZ and then stained for (upper panel) the Myc epitope (red) or (lower panel) the Myc epitope (green) and endogenous ERGIC53 (red). Scale bars: 10 µm.

To assess this possibility, we first analyzed trafficking from the ER to the ERGIC using GFP-tagged ts045-VSVG. Cells were shifted from 40°C to 17°C for 1 h to allow transport from the ER to the ERGIC, and delivery to the ERGIC was monitored by fluorescence microscopy. In control cells, a substantial proportion of ts045-VSVG was delivered to the ERGIC (Fig. 3A,B). In GMAP-210-depleted cells delivery to the ERGIC was reduced, with a proportion of cells retaining an exclusively reticular ER distribution of ts045-VSVG after 1 h incubation at 17°C (Fig. 3A,B). We noticed that the ERGIC and the Golgi complex were more compact upon GMAP-210 depletion (Fig. 3A,C; Fig. 5A,C; see also Sato et al., 2015). The reasons for ERGIC and Golgi compaction remain to be determined, but gross changes in the microtubule cytoskeleton are not responsible (Sato et al., 2015). To further assess ER to ERGIC traffic, we used the method first described by Presley et al. (Presley et al., 1997). In this method, ts045-VSVG is allowed to accumulate in the ER by incubation at 40°C, the cells are incubated on ice with nocodazole (NCZ) for 15 min to depolymerize microtubules before shifting them to 31°C in the presence of NCZ to allow exit from the ER and accumulation in peripheral pre-Golgi elements. Consistent with the original report (Presley et al., 1997), accumulation of ts045-VSVG was clearly observed in peripheral ERGIC in control cells following 20 min incubation at 31°C (Fig. 3C). In contrast, depletion of GMAP-210 resulted in the reduced appearance of peripheral ts045-VSVG spots (Fig. 3C). GMAP-210 itself was present in the peripheral elements in control cells where it coincided well with ts045-VSVG and partially with ERGIC53 (Fig. 3D). Taken together, these results are consistent with GMAP-210 localization to pre-Golgi elements, most likely corresponding to the ERGIC, and indicate a role for the protein in trafficking from the ER to this compartment.

**Fig. 4.**
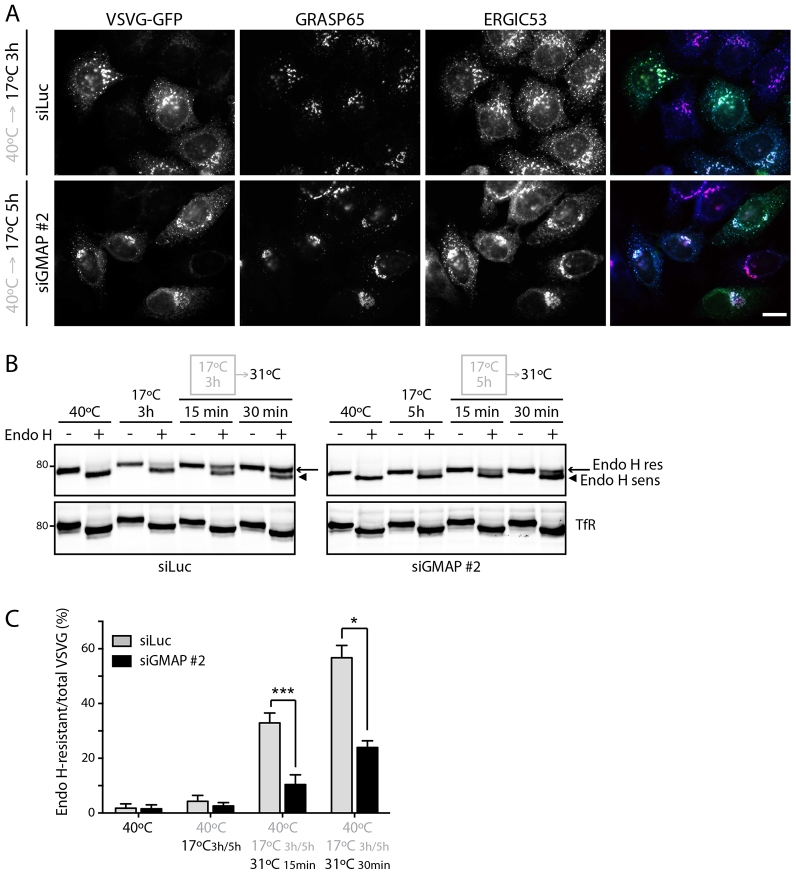
**Depletion of GMAP-210 decreases ERGIC-to-Golgi traffic of VSVG–GFP.Depletion of GMAP-210 decreases ERGIC-to-Golgi traffic of VSVG–GFP.** (A) Luciferase (siLuc) or GMAP-210 (siGMAP #2)-depleted HeLa cells infected with ts045-VSVG–GFP were incubated at 40°C for 16 h. After a 17°C block for 3 h (siLuc) or 5 h (siGMAP #2), cells were stained for endogenous GRASP65 (also known as GORASP1) (red) and ERGIC53 (blue). Scale bar: 10 µm. (B) HeLa M cells transfected with the indicated siRNAs were infected with ts045-VSVG–GFP, and incubated at 40°C for 16 h. The cells were incubated at 17°C for 3 h (siLuc) or 5 h (siGMAP #2), then shifted to 31°C for 15 min and 30 min, and lysed. Proteins were treated with Endo H (Endo H +), and analyzed by western blotting for VSVG–GFP or endogenous TfR (control for Endo H digestion). Bands corresponding to Endo-H-sensitive (arrowhead) and Endo-H-resistant (arrow) forms of VSVG–GFP are indicated. (C) The levels of Endo-H-sensitive and Endo-H-resistant forms in B were quantified. Data show mean±s.e.m from three independent experiments. *P<0.05, ***P<0.001 (paired Student's *t*-test).

We next analyzed trafficking from the ERGIC to the Golgi complex. Although the trafficking from the ER to ERGIC was delayed in GMAP-210-depleted cells, prolonged incubation at 17°C successfully led to the accumulation of the majority of ts045-VSVG in the ERGIC (Fig. 4A), allowing us to study the role of GMAP-210 in ERGIC to Golgi trafficking. After accumulation of ts045-VSVG in the ERGIC, cells were shifted to 31°C to allow delivery to the Golgi complex, which was measured by the acquisition of Endo H resistance. As shown in Fig. 4B,C, depletion of GMAP-210 slowed transport of ts045-VSVG from the ERGIC to the Golgi complex. Taken together, our results demonstrate that GMAP-210 depletion causes a delay in trafficking both from the ER to the ERGIC and from the ERGIC to the Golgi complex.

### GMAP-210 functions in Golgi to ER retrograde trafficking

**Fig. 5.**
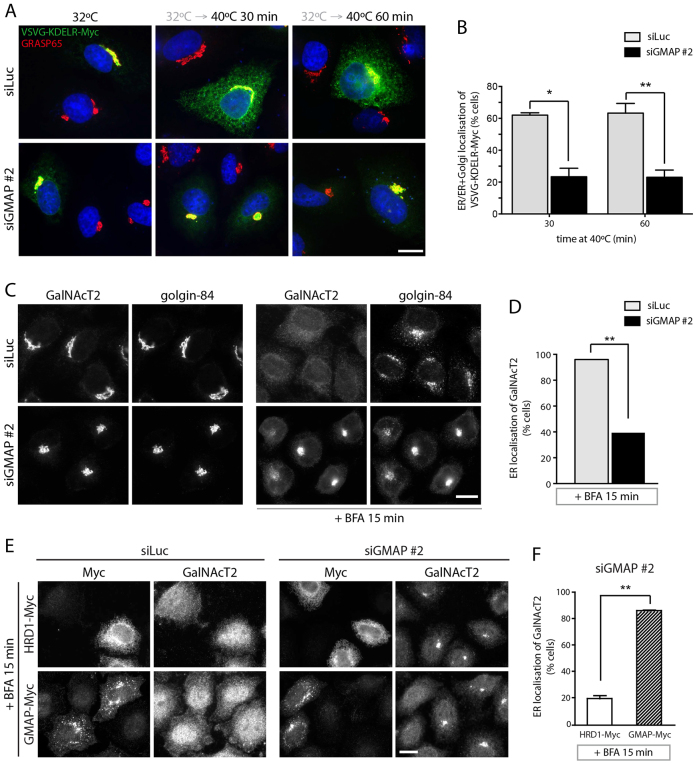
**GMAP-210 is required for Golgi-to-ER retrograde trafficking.GMAP-210 is required for Golgi-to-ER retrograde trafficking.** (A) ) Luciferase (siLuc) or GMAP-210 (siGMAP #2)-depleted HeLa cells were transfected with ts045-VSVG-KDELR–Myc and incubated at 31°C for 16 h. Cells were shifted to 40°C for the indicated time, and stained for the Myc epitope (green) and endogenous GRASP65 (red). (B) Quantification of the localization patterns of VSVG-KDELR–Myc-expressing cells (30–50 cells per condition) from A. (C) Redistribution of endogenous GalNAcT2 (green) and golgin-84 (red) from the Golgi to the ER and Golgi remnants, respectively, upon addition of 5 µg/ml BFA for 15 min was observed in HeLa cells depleted of luciferase or GMAP-210. (D) The percentage of cells displaying ER-localized GalNAcT2 after BFA treatment was determined (*n*≥100 cells per condition from three experiments). (E) HeLa cells treated with siLuc or siGMAP #2 were transfected with Myc-tagged HRD1 (control) or siRNA-resistant Myc-tagged GMAP-210. Cells were either left untreated or treated with 5 µg/ml BFA for 15 min before staining for Myc and endogenous GalNAcT2. (F) Quantification of the rescue phenotype (50–60 cells per condition) from E. Data in B, D and F show mean±s.e.m from three independent experiments. *P<0.05, **P<0.01 (paired Student's *t*-test). Scale bars: 10 µm.

GMAP-210 is known to interact with Rab2 (Sato et al., 2015; Sinka et al., 2008), which has been implicated in both anterograde ER to Golgi trafficking and retrograde trafficking back to the ER (Tisdale, 1999; Tisdale, 2000). To assess whether depletion of GMAP-210 affects COPI-dependent retrograde transport, we tracked the redistribution of a chimeric KDEL receptor (KDELR) from the Golgi to the ER (Cole et al., 1998; Yang et al., 2005). This chimera consists of the luminal domain of ts045-VSVG attached to the N-terminus of the KDELR. At the permissive temperature (31°C), this construct undergoes constitutive cycling between the ER and Golgi complex, with a predominant localization at the cis-Golgi (Fig. 5A). Upon shifting to non-permissive temperature (40°C), the chimera is retained in the ER due to ts045-VSVG misfolding, resulting in redistribution to this compartment. Appearance of ts045-VSVG-KDELR in the ER can therefore be used as a measure of retrograde trafficking. We found that ∼60% of control cells had a pronounced ts045-VSVG-KDELR localization to the ER after incubation at the non-permissive temperature, whereas in GMAP-210-depleted cells, this figure was reduced to ∼20% (Fig. 5A,B). This result indicates a defect in Golgi to ER retrograde trafficking upon GMAP-210 depletion.

To further investigate retrograde trafficking, we used BFA, which induces rapid relocation of Golgi enzymes to the ER through COPI-independent retrograde transport (Lippincott-Schwartz et al., 1990). When cells treated with control siRNA were incubated with BFA, the Golgi enzyme GalNAcT2 redistributed to the ER in 15 min, whereas the majority of GMAP-210-depleted cells retained GalNAcT2 in the Golgi (Fig. 5C,D; see also supplementary material Fig. S2). This effect was rescued by expression of siRNA-resistant GMAP-210 in the GMAP-210-depleted cells, confirming its specificity (Fig. 5E,F). These results support a role for GMAP-210 in retrograde trafficking from the Golgi apparatus to the ER.

### GMAP-210 and GM130 function redundantly in anterograde ER to Golgi trafficking

**Fig. 6.**
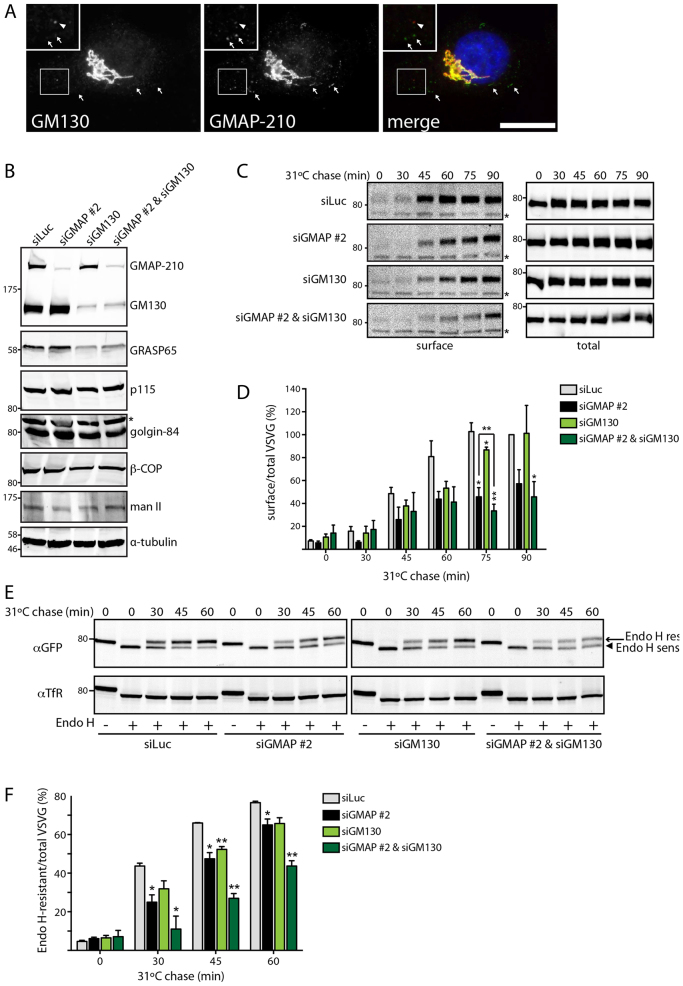
**Partial redundancy of GMAP-210 and GM130 in secretory trafficking.Partial redundancy of GMAP-210 and GM130 in secretory trafficking.** (A) HeLa cells were stained for endogenous GMAP-210 (green) and GM130 (red) followed by immunofluorescence microscopy. The insets show magnified views of the selected areas. Arrowheads indicate GM130-positive ERGIC elements with low amounts of GMAP-210 and arrows indicate GMAP-210-positive tubular ERGIC and punctate ERGIC elements lacking GM130, or with very low levels of GM130. Scale bar: 10 µm. (B) HeLa M cells transfected with the indicated siRNAs were lysed and immunoblotted for the proteins indicated. The asterisk indicates a non-specific band. (C) HeLa M cells transfected with the indicated siRNAs were infected with ts045-VSVG–GFP, and incubated at 40°C for 16 h. The cells were then shifted to 31°C for the indicated time periods (chase) to induce trafficking. Surface proteins were biotinylated and isolated using streptavidin beads. Streptavidin precipitates (surface) and total cell lysates (total) were assayed by western blotting with anti-GFP antibody. (D) The ratio of surface-to-total VSVG at the indicated time points in B is expressed as a percentage of the value in siLuc-treated cells at 90 min. Data show the mean±s.e.m from three independent experiments. (E) HeLa M cells transfected with the indicated siRNAs were infected with ts045-VSVG–GFP, and incubated at 40°C for 16 h. Cells were shifted to 31°C for the indicated time period (chase) and lysed. Proteins were treated with Endo H (Endo H +) and analyzed by western blotting with anti-GFP antibody. Bands corresponding to Endo-H-sensitive (arrowhead, ER form) and Endo-H-resistant (arrow, Golgi form) VSVG–GFP are indicated. (F) Quantification of D to indicate the percentage of VSVG–GFP in the Endo-H-resistant form. The data represent mean±s.e.m from three independent experiments. *P<0.05, **P<0.01 (paired Student's *t*-test).

**Fig. 7.**
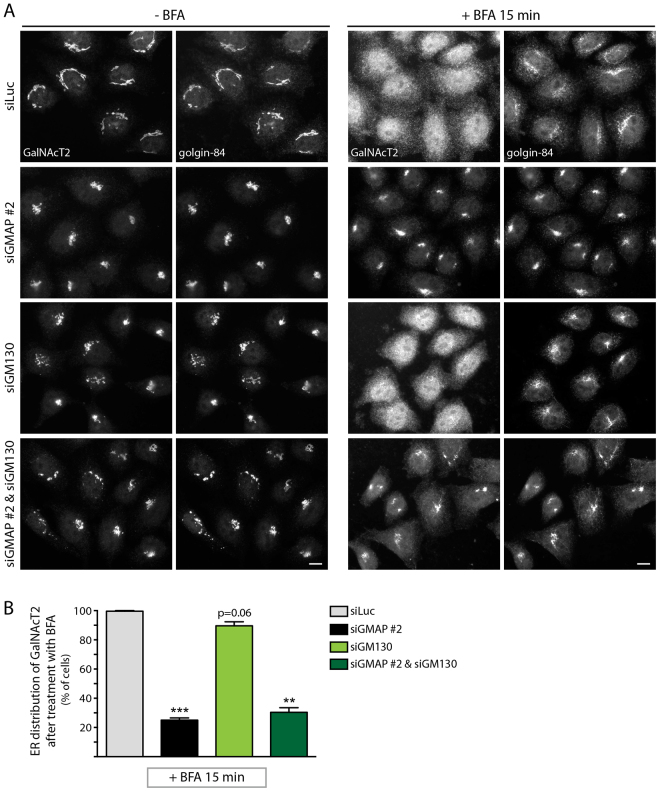
**GMAP-210 function in retrograde Golgi-to-ER trafficking is not redundant with GM130.GMAP-210 function in retrograde Golgi-to-ER trafficking is not redundant with GM130.** (A) Redistribution of endogenous GalNAcT2 and golgin-84 from the Golgi to the ER and Golgi remnants, respectively, upon addition of 5 µg/ml BFA for 15 min was observed in HeLa cells treated with the indicated siRNAs. Scale bar: 10 µm. (B) The percentage of cells displaying ER-localized GalNAcT2 after BFA treatment was determined (*n*≥100 cells per condition from three experiments). Data show mean±s.e.m from three independent experiments. *P<0.05, **P<0.01 (paired Student's *t*-test).

It has been proposed that golgins act in a redundant manner (Munro, 2011; Sinka et al., 2008). GM130, another golgin located at the cis-Golgi and ERGIC, has been implicated in ER to Golgi transport, although various different effects have been observed in different studies, with some reporting a delay in ER to Golgi trafficking upon loss or depletion of GM130 (Diao et al., 2008; Marra et al., 2007), while others reported no effect upon the rate of trafficking (Kondylis and Rabouille, 2003; Puthenveedu et al., 2006; Puthenveedu and Linstedt, 2001; Vasile et al., 2003). It is therefore possible that the role of GM130 in ER to Golgi trafficking is redundant with GMAP-210. Although both proteins are present at the cis-Golgi and ERGIC (Marra et al., 2001; Rios et al., 1994), they vary in their distribution within these compartments, with GMAP-210 predominantly localized around the rims of the cisternal region labeled by GM130 (Fig. 6A; see also Marra et al., 2001; Rios et al., 1994). GMAP-210 also appears more abundant and shares limited colocalization with GM130 at the ERGIC (Fig. 6A). To test for redundancy between GMAP-210 and GM130 in trafficking, they were depleted separately or together. Each protein was efficiently depleted alone or in combination (Fig. 6B). As shown in Fig. 6C,D, depletion of GMAP-210 or GM130 alone slowed the rate of ts045-VSVG trafficking to the plasma membrane, as detected by using surface biotinylation. The reduced trafficking of ts045-VSVG in GM130-depleted cells is in accordance with two earlier studies (Diao et al., 2008; Marra et al., 2007). Co-depletion of GMAP-210 and GM130 resulted in a marginal but significant increase in the extent of inhibition of trafficking compared to depletion of either protein alone (Fig. 6C,D). This result suggests both proteins can act in a partially redundant manner with regard to secretory trafficking. To further explore possible redundancy between GMAP-210 and GM130, ER to Golgi transport of ts045-VSVG was analyzed using EndoH resistance. Depletion of either protein alone caused an inhibition of trafficking, but the inhibitory effect was noticeably greater when both proteins were co-depleted (Fig. 6E,F). We next studied retrograde Golgi to ER trafficking, a process in which GM130 has not previously been implicated. In contrast to GMAP-210 depletion, BFA-induced retrograde Golgi to ER trafficking was unaffected by depletion of GM130 (Fig. 7A,B). Moreover, co-depletion of GMAP-210 and GM130 failed to give a stronger effect upon retrograde trafficking than depletion of GMAP-210 alone (Fig. 7A,B). Thus, GMAP-210 and GM130 are partially redundant with regard to anterograde ER to Golgi transport, but not for retrograde trafficking, which is dependent upon GMAP-210 but not GM130. GM130 therefore appears to act only in anterograde trafficking.

## DISCUSSION

Two previous studies failed to observe reduced trafficking of ts045-VSVG in cells depleted of GMAP-210 or lacking the protein altogether (Smits et al., 2010; Yadav et al., 2009), in contrast to what we report here. Both previous studies used fluorescence microscopy to monitor trafficking. Hence, the kinetic delay in trafficking we observe might have been missed in the microscopy-based approaches used earlier, which are not as sensitive as the biochemical methods employed here. It is also worth noting that the siRNA used to deplete GMAP-210 in the earlier study (designated here as siRR), gives a distinct Golgi morphology compared to the siRNAs we have used (supplementary material Fig. S3A). The broken ribbon morphology obtained with siRR is the same as that reported previously (Ríos et al., 2004; Yadav et al., 2009). This phenotype was not observed with four different siRNAs used here, which all give a compact Golgi phenotype (supplementary material Fig. S3A; see also Sato et al., 2015). The differences in morphology (and possibly trafficking) could be attributable to differences in depletion efficiency, given that siRR appears the least efficient at depleting GMAP-210 (supplementary material Fig. S3B). However, we also found that many cells transfected with siRR had dramatically enlarged LAMP-1-positive vacuoles, which were not seen with the other siRNA oligonucleotides (supplementary material Fig. S3C). This observation suggests possible off-target effects of the siRR oligonucleotide used in earlier studies (Ríos et al., 2004; Yadav et al., 2009), complicating interpretation of experiments performed with this siRNA.

Humans with mutations in GMAP-210 and GMAP-210-knockout mice die from a severe skeletal dysplasia (Smits et al., 2010). Although the mechanisms are not fully appreciated, chondrocytes from the knockout mice retain perlecan within the early secretory pathway, indicative of defective secretory trafficking of this cargo protein (Smits et al., 2010). In contrast, other extracellular matrix proteins that were analyzed were unaffected. This result indicates a cargo-specific requirement for GMAP-210 in secretory trafficking, at least within chondrocytes. A similar phenomenon was seen in our experiments, with different secreted cargoes varying in their sensitivity to GMAP-210 depletion. The same could also apply to trafficking of certain ciliary cargoes, which are particularly sensitive to loss of GMAP-210 (Broekhuis et al., 2013; Follit et al., 2008). Interestingly, although the Golgi apparatus is highly fragmented in chondrocytes, osteoblasts and kidney cells from the GMAP-210-knockout mouse, consistent with a defect in secretory trafficking, cells from the lung and intestine are unaffected (Smits et al., 2010). This indicates that different cell types display varying sensitivity to loss of GMAP-210 function. Interestingly, we could observe a similar phenomenon in cultured cells, where depletion of GMAP-210 elicited a weaker inhibition of ts045-VSVG traffic in hTERT-RPE1 cells compared to HeLa cells (supplementary material Fig. S4). Variation in sensitivity to loss of GMAP-210 between cell types could be due to differences in the secretory cargo proteins expressed in these cells, but also in the degree of redundancy between GMAP-210 and other trafficking machinery, including other golgin family members. Our finding that GMAP-210 and GM130 are at least partially redundant with regard to anterograde trafficking in HeLa cells indicates that functional redundancy between golgins can exist, as suggested previously (Munro, 2011; Sinka et al., 2008). Hence, we predict that the relative expression levels of GMAP-210 and GM130, and possibly other cis-Golgi golgins, will result in differing sensitivities to loss of function of these proteins. Moreover, we might also suppose that although golgin-mediated tethering is not essential, it increases the efficiency of trafficking, which could explain why certain cargoes or cell types with a high rate of flux through the secretory pathway are particularly sensitive to loss of golgin function. For particular cargoes, such as components of the extracellular matrix that are typically large, abundant and complex, efficiency of trafficking is likely to be more important than for simpler or less abundant cargo proteins, perhaps explaining the poor trafficking of perlecan in chondrocytes of GMAP-210-deficient mice (Smits et al., 2010).

Additional support for GMAP-210 involvement in secretory trafficking comes from overexpression studies in human and *Drosophila*, where trafficking is strongly inhibited by GMAP-210 overexpression (Friggi-Grelin et al., 2006; Pernet-Gallay et al., 2002). Too much GMAP-210, as well as too little, is therefore detrimental for trafficking, which is possibly mediated by titrating important binding partners such as ARF1 and Rab2 (Gillingham et al., 2004; Sato et al., 2015; Sinka et al., 2008). Although yeast mutants lacking the GMAP-210 homolog, Rud3, appear to grow normally, there are genetic interactions with components of the secretory trafficking machinery (Gillingham et al., 2004; Kim et al., 1999; VanRheenen et al., 1999), consistent with a conserved trafficking function for GMAP-210 across all eukaryotes.

Our results support a role for GMAP-210 in tethering at both the ERGIC and cis-Golgi, which is required for efficient trafficking in the both anterograde and retrograde directions. GMAP-210 is located at both compartments, and we observed impaired trafficking from both the ER to the ERGIC and from the ERGIC to the cis-Golgi in GMAP-210-depleted cells. We also observed a defect in retrograde trafficking from the Golgi to the ER. The latter could reflect reduced tethering of retrograde carriers, likely at the ERGIC, that are en route to the ER. Alternatively, tethering at the ER itself could be impaired, but this would seem unlikely. Also, it cannot be ruled out that GMAP-210 plays a role in the formation or movement of retrograde carriers. Indeed, BFA-induced retrograde movement of Golgi proteins to the ER is microtubule dependent (Lippincott-Schwartz et al., 1990), and previous work has suggested involvement of GMAP-210 in linking the Golgi to microtubules (Ríos et al., 2004). Further studies will be required to determine the mechanisms by which GMAP-210 contributes to Golgi to ER retrograde trafficking. Interestingly, Rab2 is also localized to both the ERGIC and Golgi complex (Tisdale and Balch, 1996), and several studies have shown it functions in both anterograde and retrograde trafficking between the ER and Golgi complex (Tisdale, 1999; Tisdale, 2000). The identification of GMAP-210 as a Rab2 effector (Sato et al., 2015; Sinka et al., 2008) is therefore consistent with a role for the protein in both anterograde and retrograde trafficking. Further support for GMAP-210 involvement in both anterograde and retrograde traffic comes from the observation that it is able to tether both anterograde ER to Golgi transport vesicles and retrograde intra-Golgi transport vesicles (Wong and Munro, 2014).

Functional redundancy between GMAP-210 and GM130 is evident for anterograde ER to Golgi trafficking. This is consistent with a recent study showing that both golgins are competent to tether ER to Golgi transport vesicles (Wong and Munro, 2014). Given that GM130, like GMAP-210, is present at both the ERGIC and cis-Golgi, it could mediate tethering at both locations (Marra et al., 2001). However, it remains to be determined whether GMAP-210 and GM130 act in parallel or in tandem to promote tethering. GMAP-210 tethers vesicles through its N-terminal ALPS motif, which is absent in GM130 (Drin et al., 2008; Sato et al., 2015). Thus the recognition of vesicles that occurs during tethering by these golgins must be different. We therefore favor the idea that the two golgins act in parallel to ensure efficient cargo delivery to the Golgi. This would fit with the different localization of the two golgins within the cis-Golgi and ERGIC. The differences in specificity of recognition events during vesicle tethering, combined with differences in golgin localization presumably also explains why GMAP-210 functions in retrograde trafficking whereas GM130 does not.

## MATERIALS AND METHODS

### Reagents and antibodies

All reagents were from Sigma-Aldrich or Merck Millipore, unless stated otherwise. Antibodies used in this study were against the following proteins: GMAP-210 (rabbit polyclonal; Sigma), GMAP-210 (mouse monoclonal; BD Biosciences), GM130 (mouse monoclonal; BD Biosciences), Myc (mouse monoclonal, 9B11; Cell Signaling Technology), Myc (rabbit polyclonal; Abcam), ERGIC53 (mouse monoclonal; Enzo Life Sciences), LAMP-1 (mouse monoclonal, H4A3; Developmental Studies Hybridoma Bank), GalNAcT2 (mouse monoclonal, UH-4; gift from Henrik Clausen), TfR (rabbit polyclonal; Millipore), GRASP65 (sheep; gift from Jon Lane), β-COP (mouse monoclonal, mAD; gift from Thomas Kreis), mannosidase II (rabbit polyclonal; Chemicon), and α-tubulin (mouse monoclonal; gift from Keith Gull). Mouse anti-p115 (4H1), rabbit anti-GM130, sheep anti-golgin-84, and sheep anti-GFP were described previously (Choudhury et al., 2009; Diao et al., 2003). Sheep anti-GMAP-210 was raised against maltose binding protein fused to amino acids 1–375 of human GMAP-210 and affinity purified against the fusion protein. Fluorophore–conjugated secondary antibodies for microscopy and Western blotting were purchased from Molecular Probes and LI-COR Biosciences, respectively.

### Plasmids and oligonucleotides

The sequence encoding full-length human GMAP-210 was amplified by PCR from cDNA provided by Francis Barr (University of Oxford, UK), and inserted in frame between the *Kpn*I and *Not*I sites of pcDNA5/FRT/TO vector (Life Technologies) for insertion of a Myc-tag at the 3′-end of the sequence. GMAP-210 resistant to siGMAP #2 was generated by introducing six silent mutations by site-directed mutagenesis. HRD1–Myc was a generous gift from Stephen High (The University of Manchester, UK). GMAP-210 ON-TARGETplus human SMARTpool (pool of four siRNAs) or each individual ON-TARGETplus siRNA were from Dharmacon, and their sequences are as follows: siGMAP #1, 5′-GGAGAUAGCAUCAUCAGUA-3′; siGMAP #2, 5′-CAAGAACAGUUGAAUGUAG-3′; siGMAP #3, 5′-GGACAUUACUAAAGAGUUA-3′; and siGMAP #4, 5′-GGGCAAGACUGGAGAGUUA-3′. The siGMAP sequence by the Rios laboratory has been published previously (Ríos et al., 2004). For GM130 knockdown, the ON-TARGETplus human SMARTpool (Dharmacon) was used. Luciferase siRNA (GL2, Eurogentec, denoted siLuc) was used as a negative control.

### Cell culture and transfection

Mammalian cells were cultured in medium supplemented with 10% HyClone fetal calf serum (FCS; Thermo Scientific) and 2 mM L-glutamine at 37°C under 5% CO_2_. HeLa and HeLa M cells were grown in Dulbecco's modified Eagle's medium (DMEM), and hTERT-RPE1 cells were grown in DMEM with Ham's F12 (1∶1) supplemented with 10 µg/ml hygromycin B (Invitrogen). Transient transfection of plasmid DNA was performed using 1 mg/ml linear polyethylenimine (PEI, Sigma-Aldrich) by forming complexes of DNA–PEI at a ratio of 1∶3, and cells were typically assayed 24 h post-transfection. For siRNA transfections, ∼4.5×10^4^ cells were seeded in 3.5-cm dishes for 24 h, transfected with 20 nM siRNA duplexes using INTERFERin (Polyplus Transfection) as specified by the manufacturer, and analyzed 72 h post-transfection. For siRNA transfections, ∼4.5×10^4^ cells were seeded in 3.5-cm dishes 24 h before transfection with 20 nM siRNA duplexes using INTERFERin (Polyplus Transfection) as specified by the manufacturer. Cells were typically assayed 72 h after transfection. For the siRNA rescue experiments, cells were sequentially transfected with GMAP-210 or control siRNA, and 48 h later with DNA encoding siRNA-resistant full-length GMAP-210–Myc or HRD1–Myc. Cells were incubated for additional 24 h, and then they were trypsinised, re-seeded onto coverslips, and assayed after another 24 h.

### Western blotting

For western blot analysis, HeLa M cells were solubilized at 4°C by an 1-h incubation with Triton X-100 lysis buffer (10 mM Tris-HCl pH 7.6, 140 mM NaCl, 1 mM EDTA, 1% Triton X-100 and a protease inhibitor cocktail). Cell extracts were clarified by centrifugation, and soluble proteins were separated by SDS-PAGE and analyzed by infrared immunoblotting. The fluorescent bands were visualized and quantified on the Odyssey Infrared Imager (LI-COR Biosciences) using the software provided by the manufacturer.

### Immunofluorescence microscopy

For immunofluorescence analysis, HeLa cells growing on coverslips were fixed with 3% (v/v) formaldehyde in PBS for 30 min at room temperature, and then permeabilized with 0.1% (v/v) Triton X-100 for 4 min. Coverslips were washed with PBS, and primary and secondary antibody incubations were performed in PBS at room temperature for 1 h each. The DNA dye Hoechst 33342 was included during the incubation with the secondary antibodies. For p115 staining, cells were fixed in 100% methanol at −20°C for 4 min. Coverslips were mounted in Mowiol 4-88, and analyzed using an Olympus BX60 upright microscope equipped with a MicroMax cooled, slow-scan CCD camera (Roper Scientific) driven by Metaview software (University Imaging Corporation). Images were processed using Adobe Photoshop CS5.

### VSVG trafficking assays

Recombinant adenovirus encoding ts045-VSVG-GFP was kindly provided by Nobuhiro Nakamura (Kyoto Sangyo University, Japan). The protocol used for infection of cells has been described previously (Roboti et al., 2013). Briefly, at 48 h after transfection with siRNA, cells were washed and incubated in 0.4 ml DMEM supplemented with 5% FCS and 0.5 µl high-titer adenovirus for 1 h at 37°C in a humidified incubator. Pre-warmed DMEM supplemented with 10% FCS (1.8 ml) was added, and the cells were shifted to 40°C for 16 h before further analysis. To initiate anterograde trafficking, cells were shifted into CO_2_-independent medium (Invitrogen) containing 0.1 mg/ml cycloheximide pre-warmed to 15°C, 17°C or 31°C. The cells were incubated in a water bath at this temperature for various times, and then either analyzed by immunofluorescence microscopy or processed for surface biotinylation or Endo H digestion as previously described (Roboti et al., 2013). Briefly, cell surface proteins were biotinylated using 0.5 mg/ml sulpho-NHS-LC-biotin (Pierce) in PBS for 30 min at 4°C. After quenching with ice-cold 50 mM NH_4_Cl in PBS at 4°C for 15 min, cells were washed with ice-cold PBS and lysed in Triton X-100 lysis buffer (10 mM Tris-HCl pH 7.6, 140 mM NaCl, 1 mM EDTA, 1% Triton X-100, and a protease inhibitor cocktail). Biotinylated conjugates were affinity purified with streptavidin resin (Pierce) at 4°C for 1 h, resolved by SDS-PAGE and detected by western blotting. For assessment of Endo H sensitivity, cells were solubilized in 100 µl Triton X-100 lysis buffer, and 50 µl of the clarified lysates were incubated with 0.5 µl Endo H (5 units/ml; Calbiochem) overnight at 37°C. Samples were analyzed by western blotting. For ER exit analysis, ts045-VSVG–GFP-expressing cells were incubated at 40°C overnight, rapidly transferred into ice-cold CO_2_-independent medium containing 0.1 mg/ml cycloheximide and 1 µg/ml nocodazole (NCZ), and incubated on ice for 15 min. Next, cells were shifted to 31°C by changing into fresh CO_2_-independent medium containing 0.1 mg/ml cycloheximide and 1 µg/ml NCZ pre-warmed to 31°C, and incubated in a 31°C water bath for 20 min. Cells were then fixed and processed for immunofluorescence microscopy. To measure retrograde transport, 48 h after siRNA transfection, HeLa cells were transiently transfected with ts045-VSVG-KDELR-Myc using linear PEI and incubated at 37°C for further 24 h. Subsequently, cells were incubated at 31°C for 16 h for the permissive condition, and then shifted to 40°C in CO_2_-independent medium containing 0.1 mg/ml cycloheximide for the non-permissive condition, and analyzed by immunofluorescence microscopy. To detect ts045-VSVG-KDELR–Myc, paraformaldehyde-fixed cells were incubated with anti-Myc antibodies diluted in 0.15% (v/v) saponin for 1 h at room temperature.

### Pulse-chase analysis of protein secretion

The secretion assay was performed using a pulse–chase approach as described previously (Roboti et al., 2013). Approximately 72 h post-transfection, cells were starved in a methionine- and cysteine-free DMEM (Invitrogen) supplemented with 2 mM L-glutamine for 20 min at 37°C, and then incubated in fresh starvation medium containing 22 mCi/ml [^35^S]Met and [^35^S]Cys protein labeling mix (PerkinElmer; specific activity >1,000 Ci/mmol) for 10 min. Brefeldin A (BFA) was added at a final concentration of 5 µg/ml at 1 hour prior to the starvation and was also included throughout the starvation and radiolabeling. After washing with PBS, the cells were collected either immediately on ice or after incubation in 1 ml serum-free DMEM supplemented with 2 mM L-cysteine and L-methionine for the indicated time periods. Secreted proteins in the media were recovered by TCA precipitation, whereas cells were solubilized with Triton X-100 lysis buffer. Precipitated proteins in the media and clarified lysates were analyzed by SDS-PAGE and phosphorimaging (FLA-3000; Fuji). [^35^S]-labeled proteins were quantified using AIDA v3.52. To assess the pattern of secreted proteins, plots of intensity of [^35^S]-labelled bands versus distance were generated using the ‘plot profile’ function of ImageJ (National Institutes of Health). Briefly, a tiff image was converted into an inverted image using the ‘invert’ tool in ImageJ, and a line scan oriented perpendicular to the respective lane of the gel was obtained.

### Statistical analysis

Quantified results are expressed as the mean±s.e.m from at least three independent experiments. The statistical significance of the results was assessed by applying a paired Student's *t*-test using Prism 6 (GraphPad). *P<0.05, **P<0.01, ***P<0.001.

## Supplementary Material

Supplementary Material
