# Supplementary material for: The golgin GMAP-210 is required for efficient membrane trafficking in the early secretory pathway
Source: J Cell Sci. 2015 Apr 15;128(8):1595–606. doi: 10.1242/jcs.166710 (PMC4406126; doi:10.1242/jcs.166710)
Supplement: Supplementary Material [file supp_128_8_1595__index.html]

Supplementary Material 

# The golgin GMAP‐210 is required for efficient membrane trafficking in the early secretory pathway

## JCS166710 Supplementary Material

**Files in this Data Supplement:**

- **Supplementary Material**
