# Supplementary material for: The golgin GMAP-210 is required for efficient membrane trafficking in the early secretory pathway
Source: J Cell Sci. 2015 Apr 15;128(8):1595–606. doi: 10.1242/jcs.166710 (PMC4406126; doi:10.1242/jcs.166710)

Supplementary Figure S1. GMAP-210 depletion decreases anterograde trafficking of VSVG. (A) HeLa M cells transfected with control siRNA targeting luciferase (siLuc), siRNA against GMAP-210 (siGMAP #1), or a mixture of four independent siRNAs against GMAP-210 (siGMAP mix) were infected with ts045-VSVG-GFP adenovirus, and incubated at 40˚C for 16 h to retain misfolded VSVG in the ER. The cells were then shifted to 31˚C for the indicated time periods (chase) to induce trafficking. Surface proteins were biotinylated and isolated using streptavidin beads. Streptavidin precipitates (surface) and total cell lysates (total) were assayed by western blotting with anti-GFP antibody. (B) The ratio of surface-to-total VSVG at the indicated time points was expressed as a percentage of the value in control cells at 90 min. Data show the mean±s.e.m from seven (siLuc, siGMAP mix) or three (siGMAP #1) independent experiments. *$P<0.05$, **$P<0.01$ (paired Student's *t*-test). (C and E) HeLa M cells transfected with the indicated siRNAs were infected with ts045-VSVG-GFP adenovirus, and incubated at 40˚C for 16 h. Cells were shifted to 31˚C for the indicated time period (chase), and lysed. Proteins were treated with Endo H (Endo H +) and analysed by western blotting with anti-GFP antibody. Bands corresponding to Endo H-sensitive (arrowhead, ER form) and Endo H-resistant (arrow, Golgi form) VSVG-GFP are indicated. (D) Quantification of (C) to indicate the percentage of VSVG-GFP in Endo H-resistant form. The data show mean±s.e.m from three independent experiments. *$P<0.05$, **$P<0.01$ (paired Student's *t*-test).

Supplementary Figure S2. Golgi-to-ER retrograde trafficking in GMAP-210-depleted cells. HeLa cells transfected with siLuc or three independent siRNAs against GMAP-210 (siGMAP ♯1-♯3) were treated with 5 μg/ml BFA for 15 min, and the redistribution of endogenous GalNAcT2 from the Golgi to the ER was assessed by

immunofluorescence microscopy. Scale bar, 10 µm.

Supplementary Figure S3. Depletion of GMAP-210 with different siRNA oligos and analysis of organelle morphology. (A) HeLa cells depleted of GMAP-210 using the indicated siRNAs were labeled for endogenous GRASP65 to visualize Golgi morphology. siGMAP ♯1-♯4 correspond to four individual SMARTpool siRNA duplexes, while siGMAP RR designates a previously reported siGMAP duplex (see main text for details). (B) Western blotting of HeLa cells treated with the indicated siRNA oligos. GMAP-210 levels were quantified for the blot shown. (C) HeLa cells treated with siGMAP RR or siGMAP mix (mix of siGMAP ♯1-♯4) were labeled for the lysosome marker LAMP-1. Scale bars, 10 µm.

Supplementary Figure S4. Depletion of GMAP-210 differentially inhibits VSVG traffic in HeLa and hTERT-RPE1 cells. HeLa (A) or hTERT-RPE1 (B) cells were transfected with the indicated siRNAs, infected with ts045-VSVG-GFP adenovirus and incubated at 40˚C for 16 h. Cells were shifted to 31˚C for the indicated time period (chase), and lysed. Proteins were treated with Endo H (Endo H +) and detected by western blotting with antibodies to GFP (top) or anti-GMAP-210 (bottom). Bands corresponding to Endo H-sensitive (arrowhead, ER form) and Endo H-resistant (arrow, Golgi form) VSVG-GFP are indicated.

Supplementary Figure 1

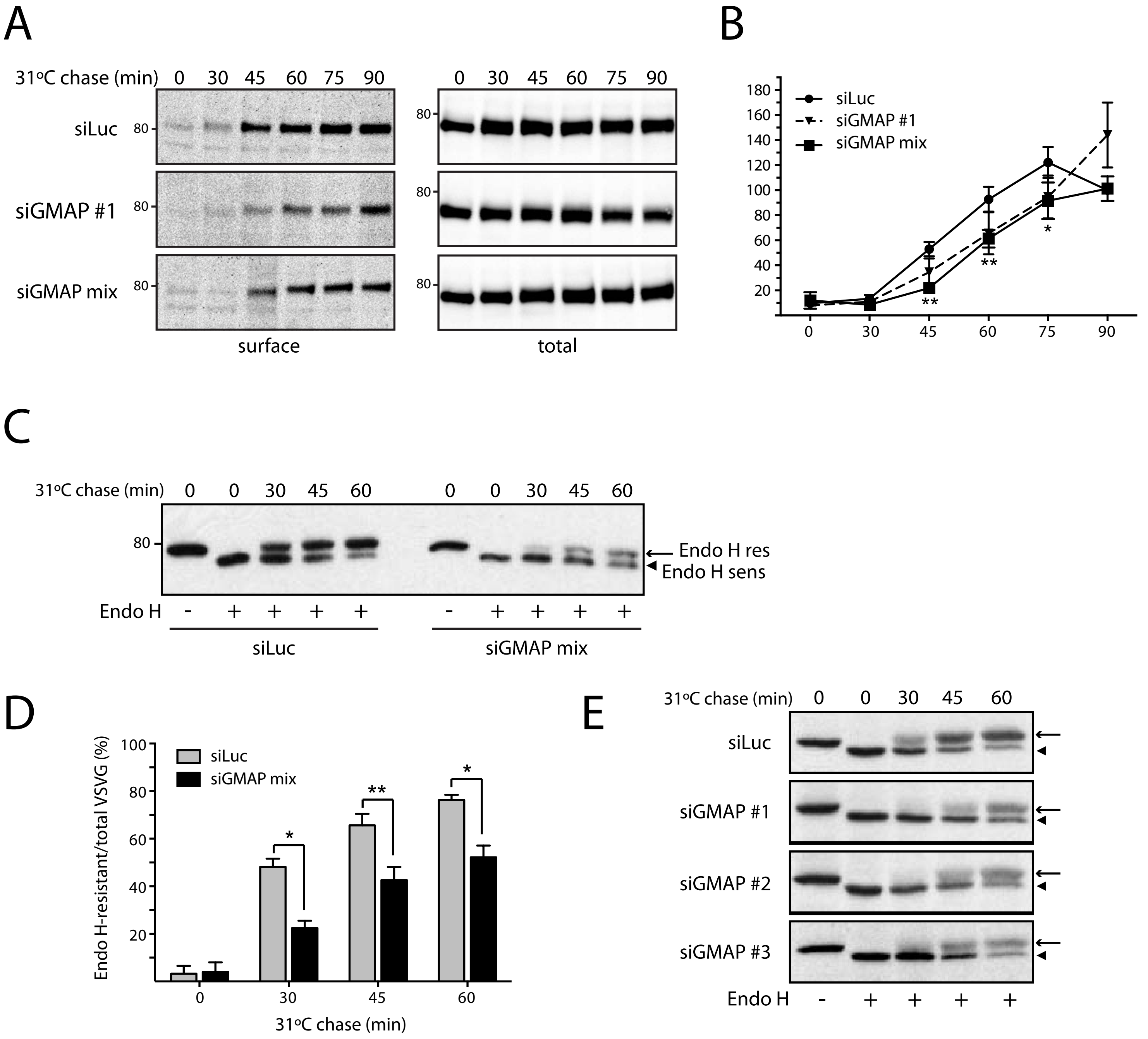

Supplementary Figure 2

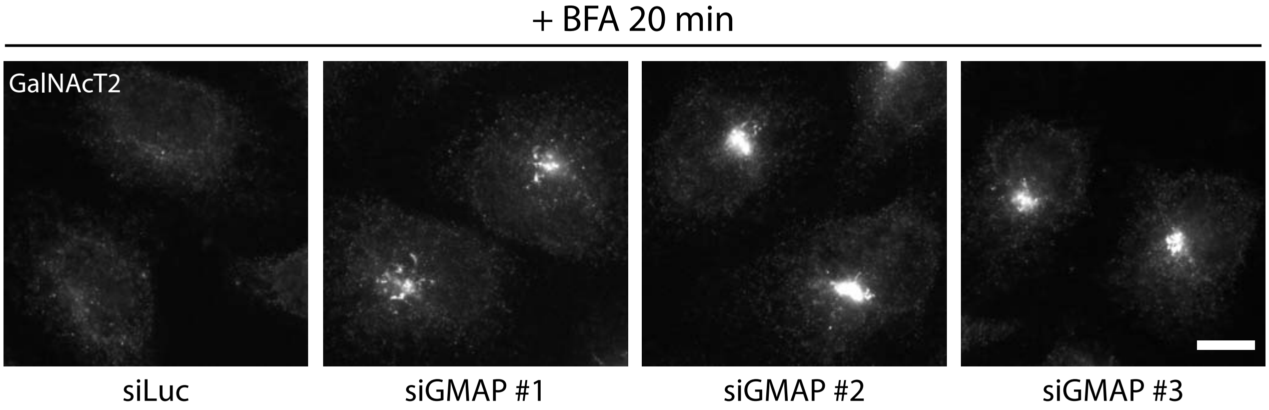

Supplementary Figure 3

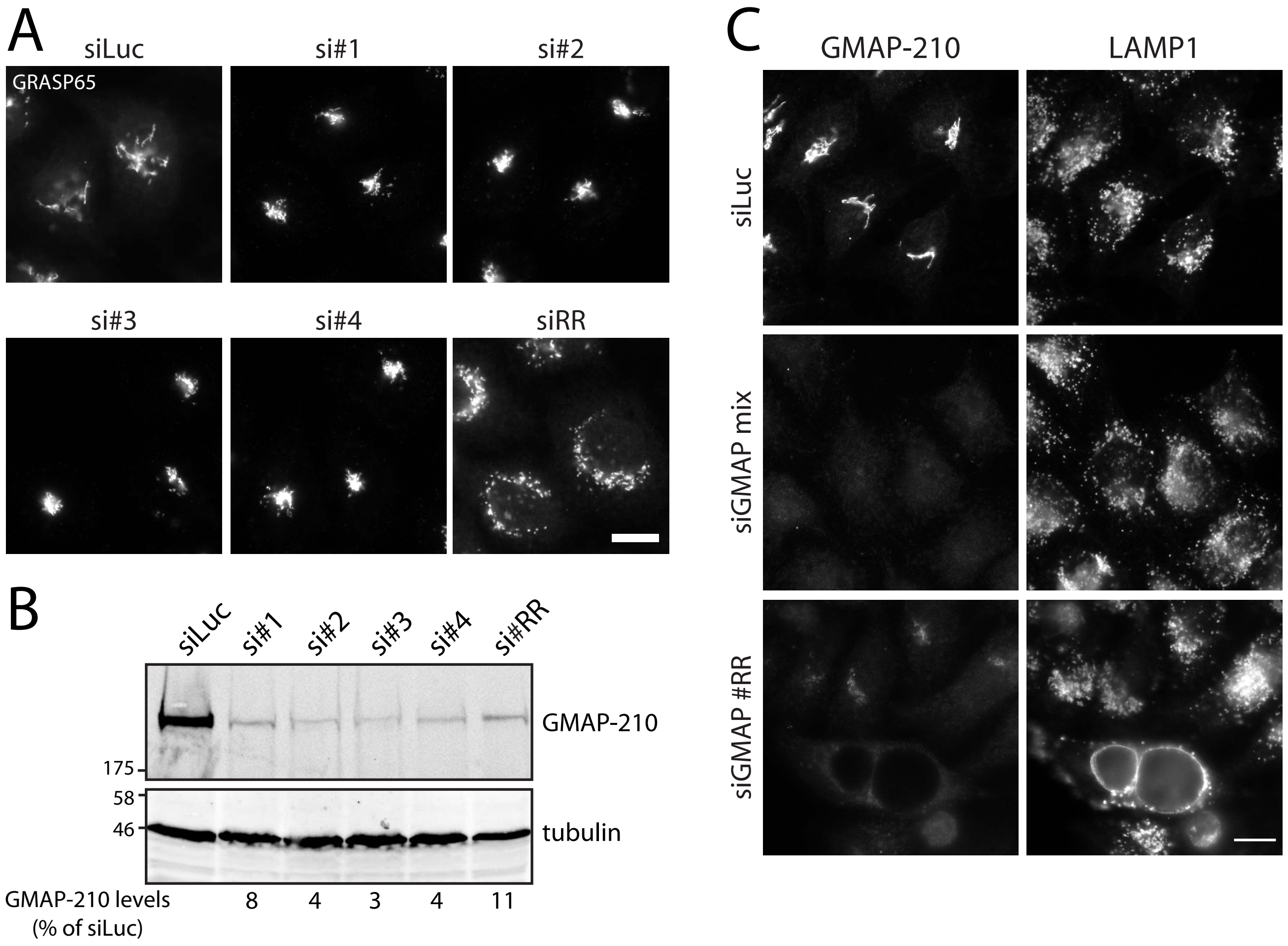

Supplementary Figure 4

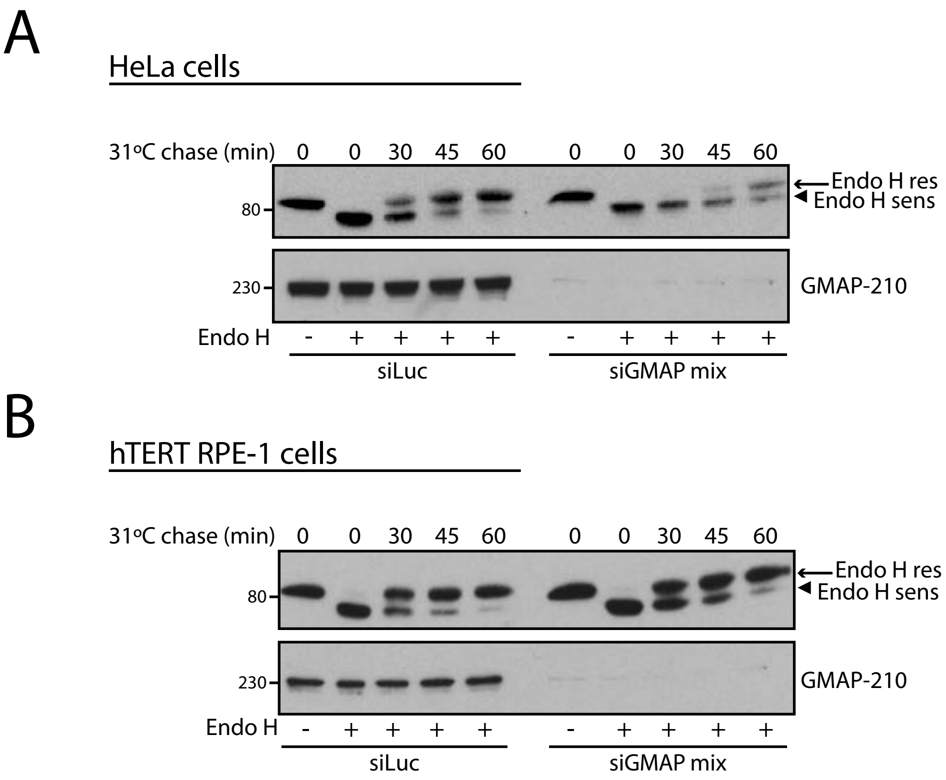

Supplement: Supplementary Material [file supp_128.8.1595_JCS166710.pdf]
